# Pulmunary artery end-diastolic forward flow as a predictor of Pulmunary valve replacement surgery complication in patients with tetralogy of fallot total correction

**DOI:** 10.1186/1532-429X-17-S1-Q92

**Published:** 2015-02-03

**Authors:** Majid Kyavar, Shabnam Madadi, Somayeh Esmaili

**Affiliations:** 1 Rajaie Cardiovascular Medical and Research Center, Iran University of Medical Sciences, Tehran, Iran (the Islamic Republic of; 2 Rajaie Cardiovascular Medical and Research Center, Iran university of medical Sciences, cardiac Electrophysiology research center, Tehran, Iran (the Islamic Republic of; 3 Cardiovascular Intervention Research Center, Rajaie Cardiovascular Medical and Research Center, Iran University of Medical Sciences, Tehran, Iran (the Islamic Republic of

## Background

Diastolic dysfunction may be initiated before systolic dysfunction and may help in early diagnosis of ventricular dysfunction.

Restriction in right ventricular fulfill status may be presented as end-diastolic forward flow (EDFF) in pulmonary artery. that may be detected in MRI by flow-sensitive phase encoding and is indicator of RVEDP. It has been demonstrated that patients with EDFF would have shorter PI and less cardiomegaly and better exercise tolerance that are all protective against right ventricle fulfilling restriction.

In patients with less EDFF, the tricuspid flow may be assessed by Doppler echocardiography in most cases; but in patients with PR, the fulfilling of right ventricle is made via both pulmonary and tricuspid valves and in these cases the Doppler echocardiography is not so definite indicator of right ventricle diastolic fulfilling.In these cases the MRI may be a good choice especially if it affects the prognosis and be associated with functional class.

Hence in this study, the EDFF was evaluated by MRI and its association with functional class and prognosis was evaluated.

## Methods

As a prospective Cohort Study (between 2012 till 2014) with Sample Size of 96 (61 male and 35 female) we evaluated TFTC patients reffered to CMR ward eavaluating RV size and function as being PVR candidate.We've measured EDFF befor surgery and the operation complication such as ICU stay duration, post-op arrhythmia and mortality for 1 month after using 1.5 Tesla Siemens Avante MRI.

The Analysis was done using a SPSS Version 13.0

## Results

This study was performed to determine the EDFF by MRI and the association of EDFF with functional class and prognosis in a one month follow-up.

We found that with a cut-off point of 114 cm/sec for EDFF, we may have the highest specificity for it and a medium-level sensitivity of 75% for prediction of complications in the patients. However for prediction of mortality, we found the EDFF as the factor with highest sensitivity and specificity that were 100% and 93.7%, respectively with a cut-off point of 127.5 cm/sec

The good sensitivity and specificity of MRI determined EDFF as a non-invasive non-time-consuming method may introduce this test as a valuable route of assessment in TFTC patients who are candidate for PVR. This factor may predict exactly the probability of successful outcomes after surgery

## Conclusions

Regarding the obtained results, it may be concluded that MRI-measured EDFF is a valuable tool with good prognostic ability in patients with TFTC who are candidate for PVR. Accordingly, use of this indicator for outcome prediction is recommended in TFTC patients to predict surgical outcomes and selection of appropriate patients for PVR

## Funding

N/A.

